# Race, Ethnicity, Nativity and Perceptions of Health Risk during the COVID-19 Pandemic in the US

**DOI:** 10.3390/ijerph182111113

**Published:** 2021-10-22

**Authors:** Thomas Jamieson, Dakota Caldwell, Barbara Gomez-Aguinaga, Cristián Doña-Reveco

**Affiliations:** 1 School of Public Administration, University of Nebraska at Omaha, Omaha, NE 68182, USA; dakotacaldwell@unomaha.edu (D.C.); bgomez@unomaha.edu (B.G.-A.); 2 Office of Latino/Latin American Studies and Department of Sociology and Anthropology, University of Nebraska at Omaha, Omaha, NE 68182, USA; cdona@unomaha.edu

**Keywords:** race, ethnicity, immigration, health risk, COVID-19, survey research

## Abstract

Previous research demonstrates that pandemics, including COVID-19, have disproportionate effects on communities of color, further exacerbating existing healthcare inequities. While increasing evidence points to the greater threat posed by COVID-19 to Latinx communities, less remains known about how identification as Latinx and migration status influence their perception of risk and harm. In this article, we use cross-sectional data from a large national probability sample to demonstrate a large positive association between ethnic identity and migration status and perceptions of harm from COVID-19 in the US. We find that individuals identifying as Hispanic/Latinx and first-generation immigrants report significantly greater risks of becoming infected by COVID-19 in the next three months, and dying from the virus if they do contract it. Further, subgroup analysis reveals that health risks are especially felt by individuals of Mexican descent, who represent the largest share of US Latinxs. Collectively, our results provide evidence about how the pandemic places increased stress on people from Latinx and immigrant communities relative to White non-Hispanic individuals in the US.

## 1. Introduction

The COVID-19 pandemic has caused widespread suffering and death around the world. Hundreds of millions of people have been infected by the virus, and at least 4.9 million people have died from COVID-19. However, the effects of the virus have not been equally distributed, with countries that explicitly prioritized economic incentives above public health having greater numbers of cases and fatalities [1]. Furthermore, there are widespread inequities in countries such as the United States, with the COVID-19 pandemic having disproportionate effects on communities of color, further exacerbating existing healthcare disparities [2,3,4]. Yet, while increasing evidence points to the greater threat posed by COVID-19 to Latinx communities, less is known about how identification as Hispanic/Latinx and migration status influence individuals’ perception of risk and harm.

In this article, we use cross-sectional data from a large national probability sample to demonstrate a large positive association between ethnic identity and migration status and perceptions of harm from COVID-19 in the US. Through an OLS regression of over 150,000 survey responses, we find that individuals identifying as Hispanic/Latinx and first-generation immigrants perceive themselves to be at significantly greater risk of becoming infected with the virus, and dying if they contract the virus. Collectively, our results provide evidence about how the pandemic places increased stress on people from Latinx and immigrant communities relative to non-Hispanic White individuals in the US. As a result, not only do Hispanic/Latinx individuals and immigrants face greater health consequences of COVID-19, but they also face a disproportionate mental burden.

This article proceeds in four further sections. First, we provide a potted review of the scholarly literature on public health among Latinxs and communities of color, especially during the COVID-19 pandemic. We conclude this section by introducing our hypotheses based on this literature. Second, we outline the data and methods employed to run our tests, including describing the data and variables from the Understanding America Study’s Understanding Coronavirus in America surveys run from the Center for Economic and Social Research at the University of Southern California. Next, we describe our results and their significance for the understanding of health disparities in pandemics. Finally, we summarize our results in the context of the broader literature, describe some limitations of the manuscript, and provide recommendations for future research to build on the findings of this article.

## 2. Determinants of Health Risk Perception

Public perception of health risks is influenced by multiple factors including the possible feelings of dread, comprehension of the complexity of the situation, uncertainty about its effects, familiarity with the risk, the possibility of solving the situation by oneself, perceived incentives for accuracy, and news coverage of public health threats [5,6,7]. However, as these risks are often domain-specific, people can differentiate between particular threats and outcomes in the process of assessing the nature of the risk [8]. Context also plays an important role in risk perception [8]. This is particularly important for minority populations in the US. Following Ferrer and Klein, we can argue that racists and xenophobic attacks on African Americans and US Latinxs can influence risk perceptions, increasing pessimistic feelings toward the possible effects of threats.

Pessimistic feelings towards possible effects of threats are furthered evidenced by Martinez Tyson, Arriola, and Corvin’s [9] research which finds that Latinx individuals across certain subgroups have comparable perceptions of risks posed particularly by mental health symptoms and diseases. Martinez Tyson and colleagues [9] also mention that economic and social discrimination could be responsible for a lack of healthcare visits by Latinx community members even though they accurately perceive symptoms and risks of diseases. Meanwhile, Bucay-Harari et al. [10] suggest that there may be a correlation between Latinx individuals and more severe mental health symptoms. Following this line of thought, we could argue that Latinx individuals are more likely to have severe mental health issues that often are exacerbated by the economic and social discrimination they experience which causes them to perceive a higher risk to their own health.

First, it is important to discuss how discrimination itself has been linked to adverse mental health outcomes in order to show its effects on the Latinx community and minority communities broadly, and to paint a linkage between discrimination and perceived health risk during COVID-19. Discrimination has been shown to be a determinant of health risk perceptions among minority groups. A number of studies have linked experienced and perceived discrimination to adverse mental health outcomes which are associated with higher perceptions of health risk [11]. Thompson [12] found that experienced discrimination is related to intrusion and avoidance symptoms regarding Black Americans, an issue that could lead towards social isolation. In particular, Thompson [12] found that appraisal of the stressfulness of the discriminatory experience was associated directly with the experiences of intrusion or avoidance symptoms. Similarly, Salgado de Snyder [13] found that experiencing discrimination for being Mexican among Mexican women was correlated with higher scores in depression on the CES-D depression scale. Williams et al. [14] showed that experiencing discrimination is linked to lower levels of subjective well-being and high distress, particularly among Black Americans.

Some research has found specific variables that are linked with psychological distress in Latinx individuals and other minorities. For instance, Brown et al. [15] found that, among Black Americans in particular, financial security is correlated with lower levels of distress, age is negatively correlated with distress (younger individuals and women generally had higher levels of distress), and higher levels of formal education are negatively correlated with distress. Brown and colleagues [15] also found that higher self-reporting of discrimination was not associated with prior mental health issues and, instead, self-reported experiences of discrimination were the factor indicating adverse mental health and distress. Bucay-Harari et al. [10] have indicated that being underinsured/uninsured is an important factor in distress, anxiety, and greater severity of mental health problems, particularly in the Latinx community. Bucay-Harari and colleagues [10] also indicate that migrants and Latinx individuals are more likely to be uninsured because of increasing barriers to these individuals in accessing private or public health insurance due to immigration status, socioeconomic factors, and a lack of political representation. At the same time as Latinx individuals are more likely to have adverse health outcomes, distrust in public institutions broadly makes reaching out to people in distress due to disasters difficult [16]. 

These studies help to demonstrate that there is a certain stress or distress associated with discrimination and how it can exacerbate increasing sociopolitical barriers to healthcare that could lead to communities more likely to experience or perceive discrimination having higher perceptions of health risk during COVID-19. One way that discrimination can correlate with higher perceptions of health risk is through the concept of stigma. This concept describes the labeling of others with attributes that are devalued or discredited by those in a position of power, stereotyping such negative attributes, and using them to separate these others from the dominant group. As a result of this separation, the stigmatized group suffers from loss of status and discrimination. Direct consequences of this stigmatization are mental and physical illness at the individual level, and unequal access to healthcare and socio-economic inequality at the macro level [17,18]. 

Oaten, Stevenson, and Case [19] found that while stigma surrounding fear of disease outbreak can cause heightened hygiene and disease avoidance at first, generally stigmatization can become a barrier to health care access. Earnshaw and Chaudoir [20] find that stigmatized groups tend to have internalized senses of inferiority compared to unstigmatized groups. Fischer et al. [21] also found that stigma can cause a significant reduction in public health measure compliance or generally impede outbreak controls. 

Stigmatization also tends to affect minority groups above others. Health-related stigmas are generally found to have adverse effects on Latinx or African American adults. Darrow, Montanea, and Gladwin [22] found that perception of HIV-infection among Latinx or African American adults is correlated with having never received an HIV test. Rueda et al. [23] demonstrated that health-related stigmas also are correlated with higher levels of anxiety, stress, and avoidance strategies. Nadeem and her colleagues [24] show that this is particularly relevant for immigrant women who are more likely than non-immigrant women to report stigma-related concerns over care and particularly over mental health care. Perreira and Pedroza [25] argue that anti-immigrant sentiments can produce higher mortality, poorer self-reported overall health and mental health specially among Latinx children and adults living in mixed-status families. Finally, Faccini et al. [26] found that, generally, stigma hinders contract tracing efforts which can then exacerbate risk in stigmatized communities. With mental health distress and a lack of healthcare access prominent with both discrimination and stigma, we can begin to discuss more direct vulnerabilities that the Latinx community has in relation to COVID-19 specifically. Due to persistent systemic inequalities in the United States, minority populations tend to have disproportionately high hospitalization rates associated with COVID-19 while also being more likely to abide by regulations or change behavior to stop the spread of disease [27,28]. Olivo et al. [29] posit reasons for these communities’ high rates of contraction of infectious diseases as being economically related to the struggle to get personal protective equipment (PPE), a dominating presence in service industry jobs that are unlikely to shut down, and generally from having to take on riskier jobs in exchange for money to leverage vulnerable economic situations, and culturally related to a higher likelihood to live in multi-generational homes. Political leaders and the public often focus on cultural factors to blame vulnerable communities for disease [30,31,32]. 

The view of cultural inadequacy rather than the general inequities driving risk factors may cause majority populations to stigmatize minority populations [30]. The reality of Latinxs’ and immigrants’ specific vulnerability to COVID-19 is compounded by their higher likelihood to avoid contact with educational or health care services due to increasing number of raids, federal immigration enforcement, and immigration surveillance at all levels of government disrupting their daily lives [33,34,35].

Immigrants in general are also more susceptible to infectious disease compared to native populations [36,37]. Limina et al. [36] found that the reasons for this increased likelihood of contracting infectious disease are the socioeconomic situations in the country in which they are living. Some of these situations are exacerbated by immigration status in the host country, social exclusion, discrimination, language difficulties, gender, and access to medical services, among other things [2,36]. Distress might be higher on undocumented immigrants having to choose on a day-to-day basis between employment status, financial security, and their health and well-being [37] as legal residency status permeates immigrants’ position in a stratified system [25,34]. 

From the perspective of the state, increased discrimination toward minority groups regarding their access to health benefits also makes them more susceptible to increased negative effects in health crises. Perreira and Pedroza [25] argue that a decline in public assistance coverage increased poverty and food insecurity among immigrant households and mixed-status families, simultaneously decreasing health utilization among immigrant women and their children. 

Far less is known about the extent to which the public perceives health risks associated with public health emergencies and infectious disease outbreaks such as COVID-19 [38]. In this area, studies have primarily come from multiple global pandemics such as the SARS and avian influenza epidemics [39], the H1N1 swine flu [40,41,42,43], and the Ebola outbreak [44,45]. Most of this literature, however, relies on nationally representative and cross-sectional data, which provides challenges in analyzing minority populations such as foreign-born groups, and racial and ethnic minorities.

In this article, we build on prior research to develop a thorough understanding of the mental health toll of the COVID-19 pandemic on Latinx and migrant individuals. Given prior work that demonstrates the disproportionate stress of public health threats for the Hispanic/Latinx community and for immigrants in the United States, we expect that perceptions of risk and harm will be greater among individuals from Latinx and first-generation immigrants than non-Hispanic/Latinx and non-immigrant individuals. Building on previous work that identifies greater levels of exposure to COVID-19 risk and harm among Latinx communities than other communities, we expect this to also be reflected in greater degrees of worry about becoming infected or dying from the virus. As a result, our hypotheses are:

**Hypothesis** **1** **(H1).** *Latinx individuals report a higher chance of becoming infected and dying from COVID-19 compared to non-Latinx individuals.*

**Hypothesis** **2** **(H2).** *First-generation immigrants report a higher chance of becoming infected and dying from COVID-19 compared to non-immigrants.*

**Hypothesis** **3** **(H3).** *Latinx individuals who are also first-generation immigrants report a higher chance of becoming infected and dying from COVID-19 compared to other individuals.*

## 3. Materials and Methods

All data for this study came from the Understanding America Study’s (UAS) Coronavirus in America Survey conducted by the Center for Economic and Social Research (CESR) at the University of Southern California. We used Waves 1–25 of the Understanding COVID-19 national studies conducted between 10 March 2020 and 31 March 2021. In total, most variables in our analysis have over 140,000 observations over this entire period, although, given not all questions were asked in each survey wave and nonresponses, we end with varying numbers of observations in each model.

### 3.1. Dependent Variables

Our two dependent variables of interest reflect participants’ perception of health risks from COVID-19. Participants were asked to indicate their perceived chance of contracting COVID-19 in the next three months and their chance of dying from COVID-19 if they do get infected by the virus. For both questions, they were asked to place their risk on a scale from 0 percent to 100 percent. To address the positive-skewed distribution of both variables and make the results more substantively meaningful, we divided these variables into quintiles. 

### 3.2. Independent Variables

We have two independent variables of interest. First, we are interested in how identifying as Hispanic/Latinx affects perception of health risks from COVID-19. This was measured through a dichotomous variable where participants were coded as 1 if they identified as Hispanic/Latinx, and 0 if they did not.

Second, we expected that an individual’s immigration status would influence their perception of health risks from COVID-19. This was measured through participants’ identification as a non-immigrant (0); first-generation immigrant (immigrant who migrated to the US) (1); second-generation immigrant (US-born children of at least one foreign-born parent) (2); third-generation immigrant (US-born children of at least one US-born parent, where at least one grandparent is foreign-born) (3); or unknown immigrant status (4). Given the absence of information about how to interpret the unknown immigrant status response, we recoded this response to indicate that these are missing values, and they were excluded from our analysis. As a result, this variable ranges from 0 to 3 with each number corresponding to the generation.

### 3.3. Control Variables

We also employed a host of control variables in our analysis to control for many plausible alternative explanations for perception of health risks from COVID-19. First, it is possible that people who experience higher levels of anxiety on a daily basis perceive higher levels of risk of becoming infected or dying from COVID-19. We took advantage of a generalized measure of anxiety in the UAS survey to control for anxiety through a measure that indicates how many days the participant had felt anxious in the past two weeks, ranging from 0 (not at all) to nearly every day (3). 

Second, given prior literature on discrimination and stigma on the health of minority communities, we created a discrimination index from felt discrimination related to COVID-19. Participants were asked whether: (1) people had acted afraid of them, (2) they had received poorer service, (3) had been threatened or harassed, or (4) treated with less courtesy and respect due to others suspecting they had COVID-19. After each of these questions were recoded to become dichotomous variables (0 = no or unsure; 1 = yes), we compiled an index by adding up the total score across all four questions.

Third, given the importance of minority languages as barriers to public health services and health literacy, we included a measure of language in our models [46,47]. The survey was available for participants to complete in either English (0) or Spanish (1), so we include a dichotomous variable that captures if a participant took the survey in Spanish or not to reflect their level of comfort with completing the survey in English. Based on literature showing that infectious disease outbreaks can create disproportionate adverse effects for linguistic minorities, we accounted for Spanish as a potential limitation to properly accessing health services [46]. Our expectation was that those completing the survey in Spanish were more likely to have greater difficulty accessing public health services in the US due to many of these services not being offered in Spanish.

Fourth, it is likely that having health insurance would shape people’s perceptions of health risk during the pandemic, especially in our models relating to the risk of dying from COVID-19 if they were to contract the virus. We expected that participants with health insurance were less likely to be worried about the health risks of COVID-19, so we included a dichotomous measure of having health insurance in our models. 

Fifth, race could also play a role in perceptions of health risk during the COVID-19 pandemic. As a result, we also included a host of dichotomous control variables capturing whether a participant identifies as White, African American, Native American, Asian, or Hawaiian or other Pacific Islander. 

Finally, we included variables for household income, whether an individual is disabled, their level of education, whether they are currently employed, their gender, and their age. Table 1 presents the operationalization, coding scheme, and descriptive statistics for all variables used in this analysis.

### 3.4. Methods

Our panel data were derived from a national probability sample weighted on gender; age; whether the participant was born in the US; education; race/ethnicity; census region; whether the participant resides in an urban, rural, or mixed zip code; employment status; number of members in the household; and household income. We weighted observations for each participant by the final post-stratification survey weights relative to the survey mean as described in the dataset.

As our dependent variables were transformed into interval measures, we conducted cross sectional analysis through ordinary least squares (OLS) regression to determine the relationship between identification as Hispanic/Latinx and immigration status on perceptions of health risks from COVID-19, with the survey data weighted as described above. 

Our model can be expressed by the following:

Perceived Risk = β_0_ + β_1_ Hispanic Latinx + β_2_ Immigration Status + ϵ. (1)

An alternative approach would be to use ordered logit regression to analyze the data given we have ordinal dependent variables. While we present results from ordinary least squares regression due to its more intuitive results, we also report results from ordered logit regression in Tables S1–S4 in the Supplementary Materials. In short, our results are robust to these alternative specifications, with the main findings consistent across these models. 

## 4. Results

Collectively, the results from our analysis support our theoretical expectations that identifying as Hispanic/Latinx and as a first- and second-generation immigrant is associated with increased perceived health risks from COVID-19. However, our results also demonstrate the complexity of race, ethnicity, nativity, and risk perceptions related to health during the pandemic. Digging deeper into heterogeneity in the results provides us with insights into both the interaction between race/ethnicity and immigration on health risks, as well as important differences in the risk perceptions among different major subgroups of the Hispanic/Latinx community in the US. Just as political scientists are increasingly calling for greater attention to heterogeneity between different Hispanic/Latinx communities in politics [48,49,50,51], our results suggest this attention to heterogeneity should also guide our understanding of health risks during the pandemic.

In this section, we first present results of the perceived risk of infection before turning to the perceived risk of dying from COVID-19. We also present results from subgroup analyses, demonstrating the variation within the Hispanic/Latinx community and the need for scholars to pay close attention to different risk perceptions among these groups.

### 4.1. Perceived Risk of Infection

First, Table 2 presents results from regressions on the perceived risk of infection among participants in our panel. To our mind, there are several important takeaways from this analysis.

First, in Model 1, a model with no control variables, identifying as Hispanic/Latinx was associated with a greater perceived risk of infection. However, this association was no longer present when controlling for alternative explanations in Model 2, suggesting other variables better explain variation in perceived risk of infection. Similarly, being a first- or second-generation immigrant did not appear to be associated with a greater perceived risk of infection as seen in Models 1 and 2. These results are counter to our expectations, suggesting that identification as Hispanic/Latinx or as a first- or second-generation immigrant do not account for risk perception by themselves.

Models 3 and 4 suggest that the interaction of these factors might be associated with greater perceived risk of infection. Identifying as a first-generation Hispanic/Latinx individual was associated with an 0.191 increase in the perceived risk of infection (*p* = 0.084) compared to individuals who are neither Hispanic/Latinx nor an immigrant, although this result did not hold at standard thresholds of statistical significance at *p* < 0.05. 

It is also important to note that the results for other variables included in the models largely fall in line with what we might expect across both Models 2 and 4. For instance, we found a positive correlation between anxiety, discrimination, and taking the survey in Spanish with perceived risk of infection. It is also worth noting the size of these correlations, with Model 4 reporting a one-unit increase in anxiety associated with a 0.253 increase in perceived risk of being infected (*p* = 0.000), a one-unit increase in the discrimination index was associated with a 0.083 increase in the perceived risk of infection (*p* = 0.001) and taking the survey in Spanish was associated with a 0.539 increase in perceived risk of infection (*p* = 0.002). 

Taken together, Table 2 presents mixed results for our hypotheses, with inconclusive results about the relationship between identification as Hispanic/Latinx and as a first- and second-generation immigrant with perceived risk of infection. To better understand these results, we conducted further tests to examine differences among different ethnic subgroups within individuals identifying as Hispanic/Latinx. 

Table 3 presents results from subgroup analysis that demonstrates the heterogeneity between different subgroups within the broader Hispanic/Latinx label. Model 2 demonstrates that identifying as Mexican was associated with a 0.160 increase in perceived risk of infection from COVID-19 (*p* = 0.45). However, identifying as Cuban was associated with a 0.600 decrease in the perceived risk of infection from COVID-19 (*p* = 0.008), and identifying as Central/South American was associated with a 0.280 decrease in the perceived risk of infection (*p* = 0.095). These results help to explain the findings in Table 2—there is a lot of variation within the Hispanic/Latinx community, which arguably reflects the cumbersome all-encompassing label for a diverse group of individuals. At the same time, nativity does not appear to be meaningfully associated with perceived risk of infection in Model 2. 

Delving deeper into the data, Models 3 and 4 show the interaction between ethnicity and migration. Again, these results demonstrate the complexity of identity and perceived risk of infection during the pandemic, with individuals identifying as first-generation Mexican associated with a 0.439 greater perceived risk of infection (*p* = 0.000) and those identifying as first-generation other Spanish individuals associated with a 0.678 increase in perceived risk of infection (*p* = 0.003) compared to individuals not identifying as Hispanic/Latinx or as an immigrant.

Finally, Table 3 reports similar findings to Table 2 in illustrating the positive relationship between anxiety, discrimination, and taking the survey in Spanish with perceived risk of infection from COVID-19. These results are consistent across Models 2 and 4, and the size and statistical significance of the results mirror those presented in Table 2, illustrating the importance of anxiety, discrimination, and taking the survey in Spanish on how individuals perceive the risk of becoming infected from COVID-19. 

### 4.2. Perceived Risk of Dying

While it is important to understand individuals’ perceived risk of infection, the COVID-19 pandemic has been particularly lethal for people of color. As a result, we turn next to analysis of individuals’ perceived risk of dying from COVID-19 if they contract the virus to see whether there are systematic differences in perceived risk related to mortality from the virus.

Table 4 presents the results of models examining the relationship between identification as Hispanic/Latinx and migration on perceived risk of dying. Results from Model 2 with all covariates included show that identifying as Hispanic/Latinx was associated with a 0.256 increase in perceived risk of dying of COVID-19 (*p* = 0.001). Consistent with earlier results, no generational nativity status was statistically different from not being an immigrant. 

Turning to Model 4, the interaction between Hispanic/Latinx and nativity appears to be positively correlated with perceived risk of dying. Identification as a first-generation Hispanic/Latinx individual was associated with a 0.262 increase in perceived risk of dying (*p* = 0.018), while identifying as a second-generation Hispanic/Latinx was associated with a 0.324 increase in perceived risk of dying from COVID-19 (*p* = 0.000) relative to non-Hispanic and non-immigrant individuals. In short, it appears that the combination of being Hispanic/Latinx and a first- or second-generation immigrant is related to increased perceived risk of dying from COVID-19.

As with earlier models, results relating to our control variables largely fall in line with what one might expect, with anxiety, discrimination, Spanish language, and age associated with greater perceived risk of dying from COVID-19 if they contract the virus. Similarly, factors that might reduce health risks were associated with decreased perceived risk of dying, such as having health insurance, identifying as White, greater household income, and education. Identifying as male was also associated with decreased perceived risk of dying.

However, it is important to further analyze the perceived risk of dying by subgroup. Table 5 presents the results of this analysis. First, we found a large degree of heterogeneity in the results, with large variations in the relationships between subgroups and perceived risk of dying. Identifying as Mexican was associated with a 0.340 increase in perceived risk of dying from COVID-19 (*p* = 0.000) and identifying as Other Spanish increased perceived risk of dying by 0.311 (*p* = 0.050). Like with previous models, nativity alone did not appear to be related to perceived risk of dying.

However, when turning to the interaction between ethnic group and immigration, we found that first- and second-generation Mexican individuals reported an increased risk of dying, by 0.541 (*p* = 0.000) and 0.377 (*p* = 0.001), respectively. Similarly, third-generation Central/South American individuals were associated with a 1.629-fold increase in perceived risk of dying. The lower self-reported risk among first- and second-generation Mexicans might be explained by Latinxs individuals who feel closer to the “canonical immigrant” tending to underreport distress as a way of defying stereotyping [52,53]. As with previous models, we found that our control variables report results in line with how one might expect them to be related to perceived risk of dying from COVID-19.

Overall, while controlling for alternative explanations, identification as Hispanic/Latinx and being first-generation was associated with greater perceived health risks from COVID-19. This was particularly true of participants of Mexican descent. However, these results are complex, and speak to the importance of treating heterogeneity among the Hispanic/Latinx community seriously in social science and public health research. We also found that anxiety, discrimination, and taking the survey in Spanish were also consistently positively associated with both the perceived risk of becoming infected and the perceived risk of dying from COVID-19. In the next section, we discuss the implications of these results.

## 5. Discussion

The COVID-19 pandemic has affected people around the world, but its effects have been particularly acute for people of color, exacerbating inequities in healthcare that existed prior to the pandemic. In this article, our findings demonstrate that people identifying as Hispanic/Latinx, and as first-generation immigrants perceive a greater likelihood of getting infected and of dying from COVID-19 than other individuals. 

Furthermore, we find important differences between different subgroups, with individuals identifying as Mexican reporting greater perceived health risk than other subgroups within the Hispanic/Latinx community. Collectively, these results build on our understanding of perceived health risks during COVID-19, demonstrating the increased perceived risks of the Hispanic/Latinx community in the US, and of first-generation immigrants especially. Building on previous studies, our results also indicate that anxiety, discrimination, and completing the survey in Spanish are also correlated with greater perceived risks of becoming infected and of dying from COVID-19. Taken together, our results add to the collective understanding of migration and public health, illustrating how ethnic identity and migration status influence individuals’ perceptions of risk during public health emergencies, including COVID-19.

While this study focuses exclusively on perceived risks and not realized health effects of the pandemic among participants in the study, these findings are significant for several reasons. First, it is likely that risk perceptions affect people’s behavior, including the adoption of protective behaviors, whether people remain in the labor force, and where people live [54,55]. Risk perceptions could exacerbate existing problems and induce the cycle of harm. For one example, perceived risks could be associated with vulnerable workers continuing to work at meatpacking plants in Nebraska where social distancing measures were insufficient, or the underground economy where lawful or safe employment is not possible [32,56]. 

Of course, workers at meatpacking plants and in other workplace environments that are particularly conducive to the spread of COVID-19 may have few alternative options for employment [57]. Further, an individual may not feel they have much choice but to work in an environment where there may be a heightened risk of exposure to COVID-19 because they need the income regardless of how they feel about the risks from the virus, and this is a very different calculation than a discretionary decision to drink at a bar. Understanding these nuances will be critical in future research to building a thorough understanding of risk perception, attitudes, and behavior in the COVID-19 pandemic. 

In particular, further research should examine the relationship between perceived risks and behavior during the pandemic, especially among the Hispanic/Latinx and immigrant communities, to better understand the adoption of protective behaviors and continued employment, especially in essential services, and how it affects internal and external migration. 

Second, building on the findings of this study, there remains scope for further exploring the mental health burden of the COVID-19 pandemic on Hispanic/Latinx and immigrant individuals. This is especially critical given the extended duration of the pandemic, and the fact that the long-term implications of the pandemic on individuals’ health, society, and the economy remain unknown at the time of writing. 

Third, a broader implication of our results is that the pandemic may only amplify existing stresses felt by Hispanic/Latinx and immigrant individuals in the US, creating a dual crisis. A heightened anti-immigration climate may increase the chronic fear of deportation, which in turn “may exacerbate current health conditions while increasing vulnerability to others” [58] p. 592. In a pandemic, Latinx communities are likely to be impacted more as they are more vulnerable to sickness and can be afraid of going to health centers which, in the case of a communicable disease, makes it more difficult to maintain public health. The role of community advocates and the separation of ICE from local police are important in reducing fear from deportation and thus allowing immigrants to have better access to health [34]. Further research should build on this study to further examine the relationship between perceived health risks of COVID-19 and the broader social and political environment in the US and its hostility to Hispanic/Latinx individuals.

It is also important to note that there are also some limitations of this study that future scholarship could address. First, our study raises important questions about the disproportionate effect of COVID-19 on at-risk populations in the United States. Unfortunately, we were unable to examine perceptions of risk with realized health effects in this article given the nature of the study, but this presents an important avenue for future research to examine. Scholars could examine the extent to which individuals’ expectations about becoming infected and dying from COVID-19 matched data about the prevalence and impact of the virus.

Second, the purpose of this study is to understand perceived risks about COVID-19 among the US population and to examine how race, ethnicity, and nativity influence risk perception. However, we are unable to speak directly to what drives our results. It is possible that some populations worry more (or less) about COVID-19 due to dissociation from the crisis, fatalistic attribution, or other factors that were not measured in the data we used. We hope that further studies examine more of the causal mechanisms associated with perceived risks among different groups.

Third, studies could also examine how individuals responded to perceived risk in their behavior regarding COVID-19. For instance, higher risk perceptions regarding becoming infected and dying from COVID-19 could be associated with greater adoption of preventive behavior to reduce the risk of becoming infected. Alternatively, individuals with higher levels of perceived risk could adopt fatalistic attitudes about the virus and become more risk-accepting. Further research should build on this study to examine the consequences of risk perception about COVID-19 on behavior.

Fourth, while this article used panel data from a national probability sample, it is observational data; therefore, causal inferences are difficult to establish. As a result, our findings should be interpreted as correlational and not causal in nature. Further work should establish the causal mechanisms through which individuals report higher levels of perceived risk relating to becoming infected and dying from COVID-19, and longitudinal studies might shed some light on these processes. This is especially important because it is possible that there is a complex causal pathway from ethnic identification and nativity to perceived risks, and anxiety, discrimination, and other factors might serve as mediating or moderating variables through this process. Future scholarship should explore these complex relationships in greater detail and establish causal pathways leading to heterogenous perceptions of risk relating to COVID-19. 

Finally, we are constrained in our analysis by the classification of the Hispanic/Latinx subgroups in the dataset, and these groups are not as specific or fine-grained as we would like. For example, it is difficult to interpret the ‘other Spanish’ classification in the data, and it would be a good practice to have additional follow-up questions to have a better understanding of the participants identifying as ‘other Spanish.’ We hope that future studies might have better data, but in our opinion this limitation further emphasizes the importance of understanding heterogeneity within racial and ethnic groups more broadly. We hope that datasets will have more fine-grained data along these lines in the future so that scholars can use them to better understand people’s experiences of COVID-19.

## 6. Conclusions

In this article, we demonstrated the positive relationship between ethnic identity and immigration status on perceptions of harm from the COVID-19 virus in the United States. Hispanic/Latinx and first-generation immigrants reported higher perceived risk of becoming infected and dying from the virus. Further analysis illustrated that this association was especially true of individuals of Mexican descent in our sample, while other Hispanic/Latinx subgroups report mixed results. Further, we found that anxiety, discrimination, and taking the survey in Spanish were positively related to perceived risk of becoming infected and dying across all models.

Taken together, our results add further evidence about the heterogeneous impact of COVID-19 on vulnerable populations in the US. Beyond increased medical risks associated with the pandemic, these results suggest that Hispanic/Latinx individuals have a higher mental health burden than other individuals with an increased perception of health risks.

The COVID-19 pandemic has had a greater worldwide impact than other public health threats in recent memory, and its full effects are yet to be realized [1,2,3,4,59]. As such, we do not know to what extent our findings might generalize to other places, settings, and times. However, we hope this study helps build on the already-impressive scholarship on the pandemic to understand its effects on vulnerable populations and immigrant communities in the US and around the world.

## Figures and Tables

**Table 1:** Descriptive Statistics.

Variable	Coding	Observations	Mean	S.D.
Perceived Risk of Infection	1 = Low risk; 2 = Medium-low risk; 3 = Medium risk; 4 = Medium-high risk; 5 = High risk	153,741	2.815	1.295
Perceived Risk of Dying	1 = Low risk; 2 = Medium-low risk; 3 = Medium risk; 4 = Medium-high risk; 5 = High risk	153,693	2.920	1.418
Hispanic/Latinx	0 = Not Hispanic/Latinx; 1 = Hispanic/Latinx	155,692	0.152	0.359
Nativity/Immigrant	0 = Not immigrant or unknown; 1 = First-generation immigrant; 2 = Second-generation immigrant; 3 = Third-generation immigrant	151,497	0.966	1.199
Anxiety (number of days feeling anxious in past two weeks)	0 = Not at all; 1 = Several days; 2 = More than half the days; 3 = Nearly every day	153,467	1.576	0.835
Discrimination Index	Index from 0–4 (0 = No discrimination; 4 = High Discrimination)	140,791	0.079	0.430
Spanish Language (Survey)	0 = English; 1 = Spanish	155,715	0.007	0.082
Health Insurance	0 = No health insurance or unsure; 1 = Has health insurance	148,141	0.903	0.296
White	0 = Not White; 1 = White	154,542	0.830	0.376
African American	0 = Not African American; 1 = African American	154,542	0.096	0.295
Native American	0 = Not American Indian or Alaska Native; 1 = American Indian or Alaska Native	154,542	0.052	0.222
Asian	0 = Not Asian; 1 = Asian	154,542	0.068	0.252
Hawaiian/Pacific Islander	0 = Not Native Hawaiian or other Pacific Islander; 1 = Native Hawaiian or other Pacific Islander	154,542	0.018	0.132
Household Income	1 = Less than $24,999; 2 = $25,000 to $49,999; 3 = $50,000 to $74,999; 4 = $75,000 to $99,999; 5 = $100,000 to $149,999; 6 = $150,000 or more	155,366	3.182	1.650
Disabled	0 = Not Disabled; 1 = Disabled	155,628	0.084	0.277
Education	1 = Less than High School Diploma; 2 = High School Graduate; 3 = Some college, no degree; 4 = Bachelor’s degree; 5 = Master’s degree; 6 = Professional or Doctorate degree	155,673	3.358	1.134
Currently Working	0 = Not working or unsure; 1 = Currently working	155,628	0.565	0.496
Male	0 = Female; 1 = Male	155,714	0.414	0.493
Age	Numeric without decimals (range from 18–111)	155,576	51.282	16.060

**Table 2:** Perceived Risk of Infection.

	(1)	(2)	(3)	(4)
Hispanic/Latinx	0.148 *	0.067		
	(0.062)	(0.068)		
First-generation immigrant	0.012	0.067		
	(0.064)	(0.076)		
Second-generation immigrant	0.014	0.055		
	(0.060)	(0.065)		
Third-generation immigrant	−0.052	−0.025		
	(0.044)	(0.046)		
Hispanic/Latinx × First-generation immigrant			0.256 *	0.190 ^+^
			(0.103)	(0.110)
Hispanic/Latinx × Second-generation immigrant			0.145 ^+^	0.108
			(0.084)	(0.086)
Hispanic/Latinx × Third-generation immigrant			0.051	0.052
			(0.133)	(0.135)
Anxiety		0.253 ***		0.253 ***
		(0.016)		(0.016)
Discrimination Index		0.083 ***		0.083 ***
		(0.025)		(0.024)
Spanish Language		0.594 ***		0.539 **
		(0.165)		(0.172)
Health Insurance		−0.032		−0.030
		(0.055)		(0.055)
White		0.083		0.068
		(0.092)		(0.093)
African American		−0.026		−0.041
		(0.096)		(0.097)
Native American		−0.096		−0.100
		(0.094)		(0.094)
Asian		−0.076		−0.061
		(0.108)		(0.109)
Hawaiian/Pacific Islander		0.212		0.200
		(0.164)		(0.167)
Household Income		−0.040 **		−0.040 **
		(0.012)		(0.012)
Disabled		0.100		0.101
		(0.070)		(0.070)
Education		−0.012		−0.011
		(0.016)		(0.016)
Currently Working		0.075 ^+^		0.076 *
		(0.039)		(0.039)
Male		−0.057		−0.058
		(0.035)		(0.035)
Age		0.000		0.000
		(0.001)		(0.001)
Constant	2.811 ***	2.751 ***	2.816 ***	2.767 ***
	(0.022)	(0.135)	(0.022)	(0.136)
Observations	149,613	128,858	149,613	128,858
*R* ^2^	0.002	0.040	0.003	0.040

Standard errors in parentheses ^+^ *p* < 0.1, * *p* < 0.05, ** *p* < 0.01, *** *p* < 0.001.

**Table 3:** Perceived Risk of Infection, by Subgroup.

	(1)	(2)	(3)	(4)
Mexican	0.239 ***	0.160 *		
	(0.072)	(0.080)		
Puerto Rican	−0.057	−0.162		
	(0.171)	(0.176)		
Cuban	−0.504 *	−0.600 **		
	(0.230)	(0.227)		
Central/South American	−0.165	−0.282 ^+^		
	(0.165)	(0.169)		
Other Spanish	0.265 ^+^	0.179		
	(0.151)	(0.147)		
First-generation immigrant	0.053	0.118		
	(0.063)	(0.074)		
Second-generation immigrant	0.018	0.067		
	(0.060)	(0.065)		
Third-generation immigrant	−0.056	−0.031		
	(0.044)	(0.046)		
Mexican × First-generation immigrant			0.529 ***	0.438 ***
			(0.107)	(0.119)
Mexican × Second-generation immigrant			0.201 *	0.164
			(0.097)	(0.104)
Mexican × Third-generation immigrant			0.174	0.198
			(0.155)	(0.156)
Puerto Rican × First-generation immigrant			−0.337	−0.488
			(0.394)	(0.398)
Puerto Rican × Second-generation immigrant			0.135	0.143
			(0.208)	(0.207)
Puerto Rican × Third-generation immigrant			−0.125	−0.157
			(0.284)	(0.314)
Cuban × First-generation immigrant			−0.473 ^+^	−0.495 *
			(0.243)	(0.240)
Cuban × Second-generation immigrant			−0.426 ^+^	−0.281
			(0.249)	(0.234)
Cuban × Third-generation immigrant			0.000	0.000
			(.)	(.)
Central/South American × First-generation immigrant			−0.150	−0.156
			(0.208)	(0.224)
Central/South American × Second-generation immigrant			−0.244	−0.337
			(0.260)	(0.224)
Central/South American × Third-generation immigrant			1.026 *	0.766 ^+^
			(0.456)	(0.428)
Other Spanish × First-generation immigrant			0.774 **	0.680 **
			(0.257)	(0.231)
Other Spanish × Second-generation immigrant			0.183	0.157
			(0.350)	(0.325)
Other Spanish × Third-generation immigrant			−0.494	−0.524 ^+^
			(0.329)	(0.304)
Anxiety		0.254 ***		0.257 ***
		(0.016)		(0.016)
Discrimination Index		0.085 ***		0.083 ***
		(0.024)		(0.024)
Spanish Language		0.667 ***		0.561 ***
		(0.153)		(0.153)
Health Insurance		−0.029		−0.025
		(0.055)		(0.054)
White		0.122		0.091
		(0.090)		(0.091)
African American		0.019		−0.010
		(0.094)		(0.094)
Native American		−0.103		−0.109
		(0.095)		(0.094)
Asian		−0.071		−0.052
		(0.108)		(0.108)
Hawaiian/Pacific Islander		0.188		0.148
		(0.165)		(0.179)
Household Income		−0.039 **		−0.037 **
		(0.012)		(0.012)
Disabled		0.101		0.105
		(0.070)		(0.070)
Education		−0.010		−0.009
		(0.016)		(0.016)
Currently Working		0.076 *		0.075 *
		(0.038)		(0.038)
Male		−0.058 ^+^		−0.060 ^+^
		(0.035)		(0.035)
Age		0.001		0.001
		(0.001)		(0.001)
Constant	2.808 ***	2.682 ***	2.816 ***	2.706 ***
	(0.022)	(0.134)	(0.022)	(0.134)
Observations	149,613	128,858	149,613	128,858
*R* ^2^	0.006	0.044	0.011	0.048

Standard errors in parentheses ^+^ *p* < 0.1, * *p* < 0.05, ** *p* < 0.01, *** *p* < 0.001.

**Table 4:** Perceived Risk of Dying.

	(1)	(2)	(3)	(4)
Hispanic/Latinx	0.181 *	0.256 ***		
	(0.072)	(0.078)		
First-generation immigrant	0.068	0.060		
	(0.074)	(0.085)		
Second-generation immigrant	0.001	0.035		
	(0.073)	(0.074)		
Third-generation immigrant	0.091	0.017		
	(0.055)	(0.054)		
Hispanic/Latinx × First-generation immigrant			0.289 **	0.261 *
			(0.107)	(0.110)
Hispanic/Latinx × Second-generation immigrant			0.203 *	0.325 ***
			(0.096)	(0.089)
Hispanic/Latinx × Third-generation immigrant			0.149	0.222
			(0.172)	(0.152)
Anxiety		0.223 ***		0.223 ***
		(0.019)		(0.019)
Discrimination Index		0.073 **		0.074 **
		(0.025)		(0.025)
Spanish Language		0.693 ***		0.747 ***
		(0.183)		(0.183)
Health Insurance		−0.121 ^+^		−0.123 ^+^
		(0.064)		(0.064)
White		−0.207 *		−0.190 ^+^
		(0.099)		(0.101)
African American		−0.035		−0.018
		(0.103)		(0.104)
Native American		−0.031		−0.027
		(0.105)		(0.105)
Asian		0.017		0.011
		(0.113)		(0.114)
Hawaiian/Pacific Islander		0.016		0.028
		(0.164)		(0.163)
Household Income		−0.110 ***		−0.110 ***
		(0.014)		(0.014)
Disabled		0.161 ^+^		0.161 ^+^
		(0.083)		(0.083)
Education		−0.113 ***		−0.113 ***
		(0.018)		(0.018)
Currently Working		−0.119 **		−0.120 **
		(0.043)		(0.043)
Male		−0.069 ^+^		−0.068 ^+^
		(0.040)		(0.040)
Age		0.019 ***		0.019 ***
		(0.001)		(0.001)
Constant	2.890 ***	2.883 ***	2.890 ***	2.866 ***
	(0.027)	(0.147)	(0.027)	(0.147)
Observations	149,572	128,843	149,572	128,843
*R* ^2^	0.003	0.123	0.003	0.123

Standard errors in parentheses ^+^ *p* < 0.1, * *p* < 0.05, ** *p* < 0.01, *** *p* < 0.001.

**Table 5:** Perceived Risk of Dying, by Subgroup.

	(1)	(2)	(3)	(4)
Mexican	0.265 **	0.340 ***		
	(0.085)	(0.092)		
Puerto Rican	−0.034	0.015		
	(0.186)	(0.161)		
Cuban	−0.088	−0.186		
	(0.166)	(0.208)		
Central/South American	−0.093	0.034		
	(0.186)	(0.201)		
Other Spanish	0.240	0.311 *		
	(0.183)	(0.158)		
First-generation immigrant	0.095	0.092		
	(0.075)	(0.086)		
Second-generation immigrant	0.006	0.043		
	(0.074)	(0.075)		
Third-generation immigrant	0.087	0.013		
	(0.055)	(0.054)		
Mexican × First-generation immigrant			0.619 ***	0.541 ***
			(0.110)	(0.124)
Mexican × Second-generation immigrant			0.225 *	0.377 ***
			(0.112)	(0.109)
Mexican × Third-generation immigrant			0.202	0.273
			(0.197)	(0.181)
Puerto Rican × First-generation immigrant			−0.248	−0.387
			(0.436)	(0.310)
Puerto Rican × Second-generation immigrant			0.244	0.219
			(0.223)	(0.187)
Puerto Rican × Third-generation immigrant			−0.453 ^+^	−0.178
			(0.250)	(0.248)
Cuban × First-generation immigrant			0.026	−0.112
			(0.167)	(0.212)
Cuban × Second-generation immigrant			−0.320	−0.015
			(0.260)	(0.427)
Cuban × Third-generation immigrant			0.000	0.000
			(.)	(.)
Central/South American × First-generation immigrant			−0.134	−0.070
			(0.215)	(0.243)
Central/South American × Second-generation immigrant			−0.011	0.289
			(0.338)	(0.319)
Central/South American × Third-generation immigrant			1.561 ***	1.636 **
			(0.444)	(0.498)
Other Spanish × First-generation immigrant			0.366	0.432
			(0.368)	(0.285)
Other Spanish × Second-generation immigrant			0.206	0.240
			(0.421)	(0.300)
Other Spanish × Third-generation immigrant			0.199	0.136
			(0.542)	(0.426)
Anxiety		0.224 ***		0.225 ***
		(0.019)		(0.019)
Discrimination Index		0.075 **		0.074 **
		(0.026)		(0.025)
Spanish Language		0.743 ***		0.768 ***
		(0.172)		(0.173)
Health Insurance		−0.118 ^+^		−0.117 ^+^
		(0.063)		(0.063)
White		−0.176 ^+^		−0.175 ^+^
		(0.099)		(0.099)
African American		0.003		0.009
		(0.103)		(0.102)
Native American		−0.037		−0.034
		(0.105)		(0.105)
Asian		0.027		0.013
		(0.114)		(0.111)
Hawaiian/Pacific Islander		0.002		−0.000
		(0.163)		(0.164)
Household Income		−0.109 ***		−0.107 ***
		(0.014)		(0.014)
Disabled		0.163 *		0.166 *
		(0.083)		(0.083)
Education		−0.111 ***		−0.111 ***
		(0.018)		(0.018)
Currently Working		−0.118 **		−0.120 **
		(0.043)		(0.043)
Male		−0.069 ^+^		−0.072 ^+^
		(0.040)		(0.040)
Age		0.019 ***		0.019 ***
		(0.001)		(0.001)
Constant	2.887 ***	2.827 ***	2.890 ***	2.811 ***
	(0.027)	(0.148)	(0.027)	(0.148)
Observations	149,572	128,843	149,572	128,843
*R* ^2^	0.005	0.125	0.008	0.126

Standard errors in parentheses ^+^ *p* < 0.1, * *p* < 0.05, ** *p* < 0.01, *** *p* < 0.001.

## Data Availability

The project described in this paper relied on public data from survey(s) administered by the Understanding America Study, which is maintained by the Center for Economic and Social Research (CESR) at the University of Southern California. The content of this paper is solely the responsibility of the authors and does not necessarily represent the official views of USC or UAS. The collection of the UAS COVID-19 tracking data is supported in part by the Bill & Melinda Gates Foundation and by grant U01AG054580 from the National Institute on Aging, and many others. This data is publicly available here: https://uasdata.usc.edu/index.php, accessed on 19 April 2021.

## Supplementary Materials

The following are available online at https://www.mdpi.com/article/10.3390/ijerph182111113/s1, Table S1: Ordered Logit Regression: Perceived Risk of Infection, Table S2: Ordered Logit Regression: Perceived Risk of Infection by Subgroup, Table S3: Ordered Logit Regression: Perceived Risk of Dying, Table S4: Ordered Logit Regression: Perceived Risk of Dying by Subgroup.
