# Supplementary material for: Race, Ethnicity, Nativity and Perceptions of Health Risk during the COVID-19 Pandemic in the US
Source: Int J Environ Res Public Health. 2021 Oct 22;18(21):11113. doi: 10.3390/ijerph182111113 (PMC8583522; doi:10.3390/ijerph182111113)
Supplement: Supplementary file 1 [file ijerph-18-11113-s001.zip › ijerph-1321723-supplementary.pdf]

Race, Ethnicity, Nativity and Perceptions of Health Risk during the COVID-19 Pandemic in the US

Supplementary Materials

Thomas Jamieson, Dakota Caldwell, Barbara Gomez-Aguinaga, and Cristián Doña-Reveco
University of Nebraska at Omaha

October 8, 2021

1 Alternative Specifications of the Models

Table S1: Ordered Logit Regression: Perceived Risk of Infection.

	(1)		(2)		(3)		(4)	
Hispanic/Latinx	0.205*		0.091					
	(0.087)		(0.099)					
First generation immigrant	0.017		0.110					
	(0.087)		(0.106)					
Second generation immigrant	0.022		0.083					
	(0.082)		(0.091)					
Third generation immigrant	-0.063		-0.030					
	(0.059)		(0.063)					
Anxiety			0.361***				0.362***	
			(0.024)				(0.024)	
Discrimination Index			0.130***				0.129***	
			(0.039)				(0.039)	
Spanish Language			0.830***				0.753**	
			(0.246)				(0.258)	
Health Insurance			-0.045				-0.043	
			(0.081)				(0.082)	
White			0.115				0.096	
			(0.133)				(0.134)	
African American			-0.047				-0.065	
			(0.138)				(0.139)	
Native American			-0.140				-0.144	
			(0.137)				(0.137)	
Asian			-0.111				-0.090	
			(0.155)				(0.157)	
Hawaiian/Pacific Islander			0.363				0.343	
			(0.261)				(0.267)	
Household Income			-0.056***				-0.056***	
			(0.017)				(0.017)	
Disabled			0.145				0.145	
			(0.105)				(0.105)	
Education			-0.012				-0.011	
			(0.022)				(0.022)	
Currently Working			0.106+				0.107*	
			(0.054)				(0.054)	
Male			-0.070				-0.070	
			(0.049)				(0.049)	
Age			0.001				0.001	
			(0.002)				(0.002)	
Hispanic/Latinx × First generation immigrant					0.359*		0.279+	
					(0.146)		(0.162)	
Hispanic/Latinx × Second generation immigrant					0.205+		0.159	
					(0.119)		(0.124)	
Hispanic/Latinx × Third generation immigrant					0.068		0.068	
					(0.191)		(0.199)	
cut1		-1.314***		-1.256***		-1.322***		-1.275***
		(0.036)		(0.198)		(0.036)		(0.200)
cut2		-0.147***		-0.047		-0.154***		-0.065
		(0.031)		(0.196)		(0.031)		(0.198)
cut3		0.393***		0.516**		0.386***		0.498*
		(0.032)		(0.196)		(0.032)		(0.197)
cut4		2.404***		2.571***		2.398***		2.554***
		(0.039)		(0.198)		(0.039)		(0.199)
Observations	149613		128858		149613		128858	

Standard errors in parentheses
$^{+}$ $p < 0.1$, * $p < 0.05$, ** $p < 0.01$, *** $p < 0.001$

Table S2: Ordered Logit Regression: Perceived Risk of Infection by Subgroup.

	(1)	(2)	(3)	(4)
Mexican	0.331**	0.220+		
	(0.102)	(0.117)		
Puerto Rican	-0.092	-0.234		
	(0.248)	(0.262)		
Cuban	-0.623*	-0.798*		
	(0.308)	(0.316)		
Central/South American	-0.233	-0.400		
	(0.237)	(0.251)		
Other Spanish	0.375+	0.262		
	(0.221)	(0.222)		
First generation immigrant	0.074	0.180+		
	(0.086)	(0.105)		
Second generation immigrant	0.031	0.103		
	(0.082)	(0.092)		
Third generation immigrant	-0.068	-0.037		
	(0.059)	(0.063)		
Anxiety		0.363***		0.371***
		(0.024)		(0.024)
Discrimination Index		0.132***		0.128**
		(0.038)		(0.039)
Spanish Language		0.919***		0.768***
		(0.229)		(0.230)
Health Insurance		-0.041		-0.037
		(0.081)		(0.080)
White		0.167		0.125
		(0.130)		(0.133)
African American		0.016		-0.024
		(0.135)		(0.137)
Native American		-0.150		-0.155
		(0.138)		(0.138)
Asian		-0.106		-0.079
		(0.157)		(0.157)
Hawaiian/Pacific Islander		0.325		0.260
		(0.265)		(0.302)
Household Income		-0.054**		-0.051**
		(0.017)		(0.017)
Disabled		0.145		0.151
		(0.105)		(0.105)
Education		-0.010		-0.009
		(0.022)		(0.022)
Currently Working		0.107*		0.103+
		(0.054)		(0.054)
Male		-0.070		-0.070
		(0.049)		(0.049)
Age		0.001		0.001
		(0.002)		(0.002)
Mexican × First generation immigrant			0.705***	0.591***
			(0.147)	(0.171)
Mexican × Second generation immigrant			0.288*	0.247
			(0.140)	(0.152)
Mexican × Third generation immigrant			0.246	0.285
			(0.222)	(0.231)
Puerto Rican × First generation immigrant			-0.434	-0.595
			(0.499)	(0.519)
Puerto Rican × Second generation immigrant			0.193	0.222
			(0.305)	(0.315)
Puerto Rican × Third generation immigrant			-0.172	-0.243
			(0.389)	(0.444)
Cuban × First generation immigrant			-0.581+	-0.635+
			(0.328)	(0.335)
Cuban × Second generation immigrant			-0.503+	-0.333
			(0.265)	(0.272)
Cuban × Third generation immigrant			0.000	0.000
			(.)	(.)
Central/South American × First generation immigrant			-0.221	-0.228
			(0.309)	(0.353)
Central/South American × Second generation immigrant			-0.310	-0.423
			(0.346)	(0.294)
Central/South American × Third generation immigrant			1.468*	1.126*
			(0.574)	(0.558)
Other Spanish × First generation immigrant			1.258**	1.144**
			(0.470)	(0.431)
Other Spanish × Second generation immigrant			0.250	0.184
			(0.471)	(0.445)
Other Spanish × Third generation immigrant			-0.733	-0.792
			(0.539)	(0.529)
cut1	-1.312***	-1.166***	-1.329***	-1.198***
	(0.036)	(0.199)	(0.036)	(0.201)
cut2	-0.143***	0.046	-0.156***	0.017
	(0.031)	(0.197)	(0.031)	(0.199)
cut3	0.399***	0.611**	0.388***	0.584**
	(0.032)	(0.196)	(0.032)	(0.199)
cut4	2.414***	2.671***	2.411***	2.650***
	(0.039)	(0.199)	(0.039)	(0.200)
Observations	149613	128858	149613	128858

Standard errors in parentheses

$^{+}$ $p < 0.1$, * $p < 0.05$, ** $p < 0.01$, *** $p < 0.001$

Table S3: Ordered Logit Regression: Perceived Risk of Dying

	(1)	(2)	(3)	(4)
Hispanic/Latinx	0.222*	0.328**		
	(0.089)	(0.102)		
First generation immigrant	0.084	0.081		
	(0.090)	(0.110)		
Second generation immigrant	0.002	0.052		
	(0.090)	(0.097)		
Third generation immigrant	0.115^{+}	0.011		
	(0.067)	(0.069)		
Anxiety		0.296***		0.295***
		(0.025)		(0.025)
Discrimination Index		0.091**		0.092**
		(0.033)		(0.033)
Spanish Language		0.865**		0.941***
		(0.269)		(0.269)
Health Insurance		-0.136		-0.138
		(0.085)		(0.085)
White		-0.247^{+}		-0.225^{+}
		(0.127)		(0.129)
African American		-0.015		0.007
		(0.132)		(0.132)
Native American		-0.022		-0.017
		(0.136)		(0.136)
Asian		0.043		0.034
		(0.146)		(0.146)
Hawaiian/Pacific Islander		0.044		0.062
		(0.211)		(0.208)
Household Income		-0.136***		-0.136***
		(0.018)		(0.018)
Disabled		0.263*		0.262*
		(0.118)		(0.118)
Education		-0.143***		-0.144***
		(0.024)		(0.024)
Currently Working		-0.166**		-0.168**
		(0.055)		(0.055)
Male		-0.099^{+}		-0.098^{+}
		(0.053)		(0.053)
Age		0.025***		0.025***
		(0.002)		(0.002)
Hispanic/Latinx × First generation immigrant			0.349**	0.332*
			(0.126)	(0.139)
Hispanic/Latinx × Second generation immigrant			0.251*	0.423***
			(0.118)	(0.115)
Hispanic/Latinx × Third generation immigrant			0.184	0.274
			(0.218)	(0.203)
cut1	-1.174***	-1.229***	-1.175***	-1.207***
	(0.037)	(0.196)	(0.037)	(0.196)
cut2	-0.283***	-0.266	-0.284***	-0.244
	(0.034)	(0.194)	(0.034)	(0.195)
cut3	0.604***	0.714***	0.603***	0.737***
	(0.036)	(0.195)	(0.036)	(0.195)
cut4	1.383***	1.569***	1.382***	1.592***
	(0.041)	(0.195)	(0.041)	(0.195)
Observations	149572	128843	149572	128843

Standard errors in parentheses

$^{+}$ $p < 0.1$, * $p < 0.05$, ** $p < 0.01$, *** $p < 0.001$

Table S4: Ordered Logit Regression: Perceived Risk of Dying by Subgroup.

	(1)	(2)	(3)	(4)
Mexican	0.320**	0.438***		
	(0.103)	(0.120)		
Puerto Rican	-0.047	0.015		
	(0.229)	(0.215)		
Cuban	-0.082	-0.279		
	(0.179)	(0.252)		
Central/South American	-0.109	0.045		
	(0.226)	(0.257)		
Other Spanish	0.310	0.398^{+}		
	(0.235)	(0.221)		
First generation immigrant	0.116	0.124		
	(0.091)	(0.111)		
Second generation immigrant	0.010	0.064		
	(0.091)	(0.098)		
Third generation immigrant	0.112^{+}	0.005		
	(0.067)	(0.069)		
Anxiety		0.297***		0.299***
		(0.025)		(0.025)
Discrimination Index		0.093**		0.091**
		(0.034)		(0.034)
Spanish Language		0.923***		0.954***
		(0.257)		(0.258)
Health Insurance		-0.133		-0.131
		(0.085)		(0.085)
White		-0.207		-0.197
		(0.126)		(0.127)
African American		0.037		0.052
		(0.131)		(0.132)
Native American		-0.031		-0.022
		(0.136)		(0.137)
Asian		0.054		0.045
		(0.147)		(0.143)
Hawaiian/Pacific Islander		0.024		0.028
		(0.210)		(0.212)
Household Income		-0.135***		-0.132***
		(0.018)		(0.018)
Disabled		0.266*		0.269*
		(0.118)		(0.118)
Education		-0.141***		-0.140***
		(0.024)		(0.024)
Currently Working		-0.167**		-0.170**
		(0.055)		(0.055)
Male		-0.099^{+}		-0.103^{+}
		(0.053)		(0.053)
Age		0.025***		0.025***
		(0.002)		(0.002)
Mexican × First generation immigrant			0.700***	0.662***
			(0.126)	(0.153)
Mexican × Second generation immigrant			0.278*	0.499***
			(0.139)	(0.141)
Mexican × Third generation immigrant			0.251	0.342
			(0.246)	(0.239)
Puerto Rican × First generation immigrant			-0.256	-0.455
			(0.496)	(0.373)
Puerto Rican × Second generation immigrant			0.303	0.324
			(0.278)	(0.245)
Puerto Rican × Third generation immigrant			-0.469	-0.187
			(0.288)	(0.333)
Cuban × First generation immigrant			0.050	-0.197
			(0.181)	(0.257)
Cuban × Second generation immigrant			-0.247	0.094
			(0.229)	(0.455)
Cuban × Third generation immigrant			0.000	0.000
			(.)	(.)
Central/South American × First generation immigrant			-0.152	-0.050
			(0.251)	(0.300)
Central/South American × Second generation immigrant			-0.006	0.308
			(0.417)	(0.416)
Central/South American × Third generation immigrant			2.317*	2.408*
			(0.921)	(0.951)
Other Spanish × First generation immigrant			0.497	0.588
			(0.527)	(0.442)
Other Spanish × Second generation immigrant			0.253	0.271
			(0.518)	(0.378)
Other Spanish × Third generation immigrant			0.262	0.149
			(0.816)	(0.660)
cut1	-1.172***	-1.155***	-1.179***	-1.127***
	(0.037)	(0.197)	(0.038)	(0.198)
cut2	-0.281***	-0.191	-0.286***	-0.161
	(0.034)	(0.196)	(0.034)	(0.197)
cut3	0.607***	0.791***	0.605***	0.823***
	(0.036)	(0.196)	(0.036)	(0.197)
cut4	1.388***	1.647***	1.387***	1.680***
	(0.041)	(0.197)	(0.041)	(0.198)
Observations	149572	128843	149572	128843

Standard errors in parentheses
$^{+}$ $p < 0.1$, * $p < 0.05$, ** $p < 0.01$, *** $p < 0.001$
